# Physiotherapy Rehabilitation Approach for Enhancing Stability and Gait in a Patient With Cerebellar Ataxia: A Case Report

**DOI:** 10.7759/cureus.70967

**Published:** 2024-10-06

**Authors:** Devashish Thote, Vaishnavi Yadav, Nikita Bhusari, Ritik Daf, Ishika Agrawal, Sarang Bhoyar

**Affiliations:** 1 Department of Physiotherapy, Ravi Nair Physiotherapy College, Datta Meghe Institute of Higher Education and Research, Wardha, IND

**Keywords:** ataxic gait, balance, brainstem encephalitis, cerebellar ataxia, coordination

## Abstract

Brainstem encephalitis (BE), a rare and potentially fatal condition, can lead to significant neurological impairments, including cerebellar ataxia. This case report describes a 23-year-old female who presented with weakness in both upper and lower limbs, seizures, and an ataxic gait pattern following a history of sepsis-related brainstem encephalitis. Magnetic resonance imaging (MRI) revealed gross cerebellar atrophy. The patient underwent a comprehensive, goal-oriented physiotherapy protocol targeting functional mobility, strength, balance, and coordination. Interventions included task-specific exercises, resistance training, gait training, and coordination drills. Post-intervention assessments showed notable improvements in muscle tone, deep tendon reflexes, and outcome measures such as the Scale for the Assessment and Rating of Ataxia (SARA), Berg Balance Scale, and Functional Independence Measure (FIM). This case highlights the importance of early and targeted physiotherapy in managing cerebellar ataxia, demonstrating significant functional recovery and improved quality of life. The findings support the critical role of specialized rehabilitation programs in the comprehensive care of patients with cerebellar ataxia following brainstem encephalitis.

## Introduction

Brainstem encephalitis (BE) is an uncommon and potentially fatal inflammatory condition affecting the brainstem, a critical structure in the brain responsible for vital functions such as breathing, heart rate, and movements. It is a term used to describe a wide range of viral and inflammatory disorders that affect the pons, cerebellum, and medulla oblongata as part of the rhombencephalon, with varying prognoses [1]. There are a number of infectious and autoimmune causes associated with this challenging condition. Infectious brainstem encephalitis (BE) is most frequently caused by listeria. The Gram-positive, facultative, aerobic, non-spore-forming bacillus *Listeria monocytogenes* is the causative agent. Undercooked meals and unpasteurized milk are the primary oral routes of transmission [2]. Every year, encephalitis impacts one in 10000 people, and it frequently has catastrophic neurological effects [3]. Cranial neuropathies, altered states of consciousness, quadriplegia, and ataxic gait are among the clinical manifestations of brainstem encephalitis. Pathologies of peripheral nerves and damage to the central nervous system can cause ataxia. Cerebellar injury, often brought on by a stroke, illness, or tumor, is one of the most frequent causes of ataxia. In addition to the accompanying high-amplitude tremor, cerebellar damage normally causes the ataxia of voluntary movement. An ipsilateral body part will experience symptoms if just one cerebellar hemisphere is damaged [4]. The cerebellum can synchronize activity in several effectors, including the head, arm, leg, and eye [5]. Deficiencies in limb control and ocular control, for instance, while walking or reaching, covary, suggesting that similar challenges to programming coordinated actions may be at play. It follows from these interactions that when eye and limb movements are linked, as is usually the case during functional activities, their precision may be further compromised [6]. People with cerebellar ataxia suffer from dyssynergia, an impairment of multi-joint movements [7]. Cerebellar dysfunction is characterized by more variability in stride length and individual joint kinematics, extended periods in double stance, and poor coordination between the limbs [8].

Physiotherapy plays a crucial role in managing cerebellar ataxia, significantly improving balance, gait, and overall function. The purpose of physical therapy should be to help patients reach their full potential and minimize their problems throughout their illness, as it can improve the health and well-being of those suffering from ataxia [9]. The physiotherapist designed exercises that challenge and improve balance, leading to steadier walking and reducing fall risk. Specific exercises target gross and fine motor skills, retraining the brain to control the movement more precisely. Strengthening exercises for core trunk and leg muscles improve posture, stability, and endurance, allowing easier movement and reducing fatigue. It is a powerful tool for managing symptoms, improving function, and enhancing the quality of life.

## Case presentation

Patient information

A 23-year-old female came to the hospital with a complaint of weakness in both upper and lower limbs, along with seizures. Her guardian reported a previous history of abnormal movement of the limbs and body when she was diagnosed with sepsis with brainstem encephalitis with quadriplegia two years back, for which she underwent medical treatment. Now, after the postpartum period, she is complaining of weakness in both upper and lower limbs with seizures and having an ataxic gait pattern; for these, she was admitted to the Acharya Vinoba Bhave Rural Hospital medicine ward. Following the investigations, magnetic resonance imaging (MRI) showed that there was gross atrophy of the cerebellar hemisphere and vermis with the prominence of retro-cerebellar and infra-cerebellar cerebrospinal fluid (CSF) space. With this complaint, the patient was transferred to a neurology ward for further treatment.

Clinical findings

Before the examination, the patient was briefed about the process, and her consent was obtained. On the day of the assessment, the patient was in a supine position. She was conscious, oriented, and responsive to vocal commands and was of ectomorphic build. A higher mental function test was conducted; the Mini-Mental State Examination (MMSE) result was 21/30. The sensory examination showed intact sensations. During the motor examination, the tone assessment indicated the presence of grade 2+ tone (tone grading scale) in both upper and lower limbs, as shown in Table 1. An evaluation of the cerebellum and gait revealed impaired heel-to-toe and finger-to-nose test results on both sides, along with an inability to walk due to imbalance. The assessment of the deep tendon reflex is mentioned in Table 2. The muscle strength in both the upper and lower limbs was 2/5.

**Table 1:** Assessment of muscle tone by tone grading scale

Tone	Left	Right
Upper limb	2+	2+
Lower limb	2+	2+

**Table 2:** Pre-intervention deep tendon reflex

Deep tendon reflex grading	Right	Left
Biceps jerk	3+	3+
Triceps jerk	3+	3+
Supinator jerk	2+	2+
Knee jerk	3+	3+
Ankle jerk	3+	3+
Plantar response	Extensor	Extensor

Diagnostic methods

**Figure 1:**
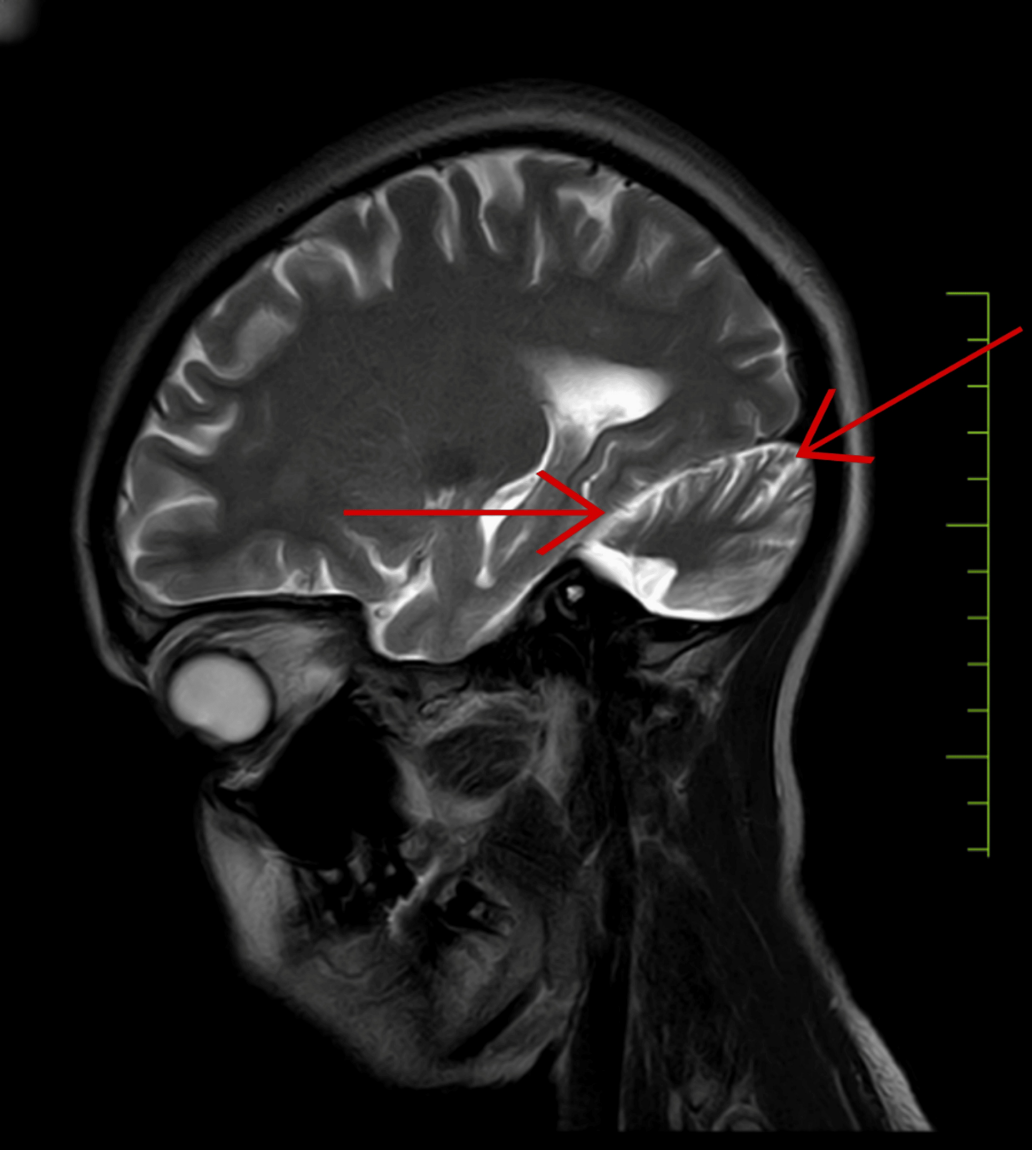
Magnetic resonance imaging (MRI) of the brain showing the atrophy of the cerebellar region

The patient underwent an investigation of magnetic resonance imaging (MRI) of the brain (Figure 1), which revealed that there is gross atrophy of the cerebellar hemisphere and vermis with the prominence of retro-cerebellar and intracerebellar cerebrospinal fluid (CSF) space and the communication of the fourth ventricle with retro-cerebellar cerebrospinal fluid (CSF) space.

Diagnostic assessment

Physiotherapy Interventions

Goal-oriented physiotherapy protocol was planned for the patient for six weeks, which is mentioned in Table 3.

**Table 3:** Goal-oriented physiotherapy protocol

Problem identified	Goals	Therapeutic interventions
Decreased functional mobility	To improve functional mobility	Task-specific exercises to improve daily activities, sit-to-stand exercises (Figure 2), marching in place, functional reach exercises, and obstacle course activities
Generalized weakness	For strengthening the upper and lower limbs	Exercises using progressive resistance. After 10 repetitions with a 1 kg weight cuff, progressively increase the weight
Difficulty walking independently	To achieve independent walking	Parallel-bar walking is done in front of a mirror
Difficulty in stair-climbing	To achieve balance in stair-climbing	The patient was instructed to walk in front of a mirror, walk parallel to bars, and climb up and down a stepper that was placed in front of him. The exercise is performed 10 times
Impaired neuromuscular control	To improve neuromuscular control	Balance board exercises and single-leg stance: stand on one leg for 30 seconds to one minute, gradually increasing the duration
Loss of balance and coordination	To improve balance and coordination	Coordination drills, gait training, and stability exercises. Frenkel's exercises

**Figure 2:**
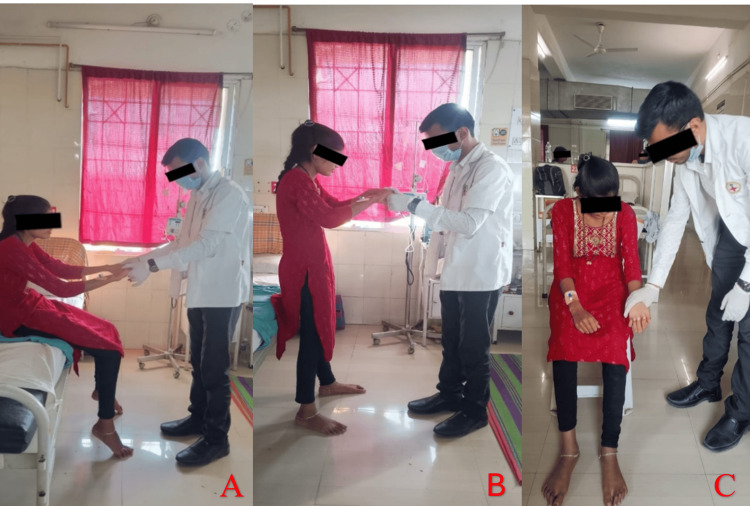
(A and B) The patient performing sit-to-stand training. (C) Upper limb strengthening with manual resistance

Outcome Measures

As further evidence of the effectiveness of the intervention, post-intervention deep tendon reflex and outcome measures were taken after six weeks as shown in Table 4 and Table 5, respectively.

**Table 4:** Post-intervention deep tendon reflex

Deep tendon reflex	Right	Left
Biceps jerk	2+	2+
Triceps jerk	2+	2+
Supinator jerk	2+	2+
Knee jerk	2+	2+
Ankle jerk	2+	2+
Plantar response	Extensor	Flexor

**Table 5:** Outcome measures

Serial number	Scales	Pre-intervention	Post-intervention
1	Scale for the Assessment and Rating of Ataxia (SARA)	25/40	22/40
2	Berg Balance Scale	18/56	29/56
3	Manual muscle testing	2/5	4/5
4	Barthel index	25/100	65/100
5	Functional Independence Measure (FIM)	68/126	98/126

## Discussion

Physical therapy should be initiated as soon as ataxia is diagnosed, even in patients who exhibit modest symptoms. According to studies, physiotherapy techniques that promote balance and independence in daily living tasks, as well as dynamic balance training, can improve gait results [10]. Ilg and colleagues reported encouraging results from physical therapy for ataxia patients. Their study demonstrated how coordination training improved motor function and reduced ataxia symptoms, helping patients achieve significant personal goals, for example, exercises to improve balance and coordination [11]. According to Milne and colleagues, a variety of rehabilitation modalities were used, including treadmill training, multimodal inpatient rehabilitation, cycling, biofeedback-assisted balancing exercises, coordination training, and balance training. According to the authors, rehabilitation improved ataxia, function, mobility, and balance continuously [12].

In addition to poor balance reactions, diminished anticipatory postural control, coordination issues, and a decreased capacity to learn from movement errors, ataxia sufferers face a wide variety of issues [13]. When the cerebellum cannot perform these functions, ataxic gait develops as a compensatory walking pattern [14]. Strength and balance training tailored to the unique challenges of each patient can be an effective therapeutic intervention [15]. As we are aware that balance and coordination exercises help ataxic patients, we need to find ways to modify patients' routines so they are engaged in ongoing, varied, and effective training; customize training to meet each patient's unique needs based on their age, interests, and stage of the disease; maintain patients intensive exercise regimens; develop long-term studies; and integrate rehabilitation into clinical practice [16,17].

Despite a low level of quality data published on the topic, most professionals agree that early rehabilitation is better for patients with ataxia over the long term [18]. Several regimens aim to enhance static and dynamic balance in individuals who are experiencing gait and balance impairments. Several changes were noted after therapy, including improvements in muscle tone, strength, mobility, and balance coordination, all of which are vital for individuals who need assistance with daily activities.

## Conclusions

A physiotherapy regimen was shown to be beneficial for patients with cerebellar ataxia in this study. In this paper, the importance of physical therapy regimens is emphasized in relation to acute cases. These treatments may prove useful in other clinical settings. A neurophysiotherapy protocol significantly improved strength, balance, and coordination. A physiotherapist's specialized rehabilitation program is an essential part of the comprehensive care neurosurgery patients receive because it can help them regain their strength, mobility, and overall well-being.
